# The Patient Experience of Prior Authorization for Cancer Care

**DOI:** 10.1001/jamanetworkopen.2023.38182

**Published:** 2023-10-18

**Authors:** Fumiko Chino, Alexandra Baez, Ivy B. Elkins, Emeline M. Aviki, Lauren V. Ghazal, Bridgette Thom

**Affiliations:** 1 Affordability Working Group, Memorial Sloan Kettering Cancer Center, New York, New York; 2 Department of Radiation Oncology, Memorial Sloan Kettering Cancer Center, New York, New York; 3 City College of New York, New York, New York; 4 EGFR Resisters, Evanston, Illinois; 5 Department of Surgery, Gynecology Service, Memorial Sloan Kettering Cancer Center, New York, New York; 6 School of Nursing, University of Michigan, Ann Arbor; 7 Department of Medicine, Memorial Sloan Kettering Cancer Center, New York, New York

## Abstract

**Importance:**

Prior authorization (PA) requires clinicians and patients to navigate a complex approval pathway. Resultant delays and denials can be particularly problematic for patients with cancer, who often need urgent treatment or symptom management.

**Objective:**

To investigate the patient perspective of PA for cancer-related care, including perceptions about the process, outcomes (including delays and denials), and patient administrative burden.

**Design, Setting, and Participants:**

This cross-sectional, anonymous survey used a convenience sample of patients with PA experience. Participants were recruited using social media and email lists of US-based cancer advocacy organizations from July 1 to October 6, 2022.

**Exposure:**

Prior authorization for any cancer-related service.

**Main Outcomes and Measures:**

Delays to care, outcome of PA, communication, and changes in anxiety (measured on a scale of 0-100, with 0 indicating no anxiety and higher scores indicating higher levels of anxiety) and trust.

**Results:**

Of 178 respondents (158 women [88%], 151 non-Hispanic White respondents [84%], 164 respondents [92%] <65 years), 112 (63%) reported that their cancer care was approved and given as recommended, and 39 (22%) did not receive recommended care due to delays or denials. Most respondents (123 [69%]) reported a PA-related delay in care; of those with delayed care, 90 (73%) reported a delay of 2 or more weeks. Most respondents (119 [67%]) had to personally become involved in the PA process; 35 (20%) spent 11 or more hours dealing with PA issues. Overall, the PA experience was rated as bad (70 [40%]) or horrible (55 of 174 [32%]); ratings were associated with the length of delay (ρ = 0.36; *P* < .001) and the time spent on PA (ρ = 0.42; *P* < .001). Self-reported PA-related anxiety was higher than usual anxiety (mean [SD] score, 74.7 [20.2] vs 37.5 [22.6]; *P* < .001) and was correlated with delay length (ρ = 0.16; *P* = .04), time spent on PA (ρ = 0.27; *P* < .001), and overall PA experience (ρ = 0.34; *P* < .001). After PA, 159 respondents (89%) trusted their insurance company less, and 148 respondents (83%) trusted the health care system less. Patient involvement in the PA process was associated with increased odds of distrusting their insurance company (β = 6.0; 95% CI, 1.9-19.2) and the health care system (β = 3.3; 95% CI, 1.4-8.1) and of having a negative experience (β = 6.6; 95% CI, 3.1-14.3).

**Conclusions and Relevance:**

This survey-based cross-sectional study of the patient experience with PA highlights an adverse outcome of PA: 22% of patients did not receive the care recommended by their treatment team because of PA. Most respondents experienced a delay in recommended oncology care, and delays were associated with increased anxiety, a negative perception of the PA process, and patient administrative burden.

## Key Points

**Question:**

What is the patient experience with prior authorization (PA) for cancer-related care?

**Findings:**

This cross-sectional study of 178 patients with cancer with experience with PA showed delays to care (with most delays ≥2 weeks), increased anxiety, and patient administrative burden. The PA process was rated bad or horrible by most respondents and was associated with decreased trust in the health care system.

**Meaning:**

This study suggests that PA for cancer care can have discrete negative associations with outcomes for patients; streamlining the process is key to optimizing the quality of care delivered and improving the patient experience with cancer care.

## Teaser

This survey-based cross-sectional study investigates the patient perspective of prior authorization for cancer-related care, including perceptions about the process, outcomes (including delays and denials), and patient administrative burden.

## Introduction

Prior authorization (PA) is a management process in which health insurance companies require clinicians to obtain approval for medical services before covering the costs. This practice purports to increase adherence to evidence-based medicine standards, limit the use of unnecessary care, and reduce health care costs. However, PA processes may require clinicians and patients to navigate a complex approval pathway and can lead to delays in receipt of care or denials of recommended treatment.^1,2,3^ Utilization management through PA has been increasing over time,^4^ and resultant delays and denials can be particularly problematic for patients with cancer, who often need urgent treatment or symptom management.^5^

Oncologists have reported suboptimal care and delays in cancer treatment owing to PA’s bureaucratic interference in clinician-patient decision-making; an oncology survey found that payer pressures, including handling PA, ranked as the most pressing practice concern.^5^ An American Medical Association survey found that 90% of respondents reported treatment delays due to PA.^6^ Although 73% of surveyed oncologists reported that patients “routinely” expressed concerns to them about PA-related delays,^7^ the experience of patients faced with a PA barrier is not well explored, to our knowledge.

The purpose of this study was to understand the patient perspective of PA, including perceptions about the PA process, outcomes (including delays and denials), and patient administrative burden. We further explored the association of PA with patient trust in their care team, insurance provider, and the broader health care system, as well as self-reported anxiety.

## Methods

A convenience sample was recruited from July 1 to October 6, 2022, using social media (Twitter [now known as X] and Facebook) and US-based cancer advocacy organizations’ email lists. Eligible participants were 18 years or older with a personal experience with PA for cancer treatment, as confirmed through an affirmative response to a screening question. Respondents answered an anonymous survey of investigator-designed questions on their PA experience, including the outcome of the PA (treatment approved and recommended care delivered; treatment approved but different care delivered because of delay; treatment not approved but recommended care delivered, with patient paying out of pocket; treatment not approved and different care delivered; or different outcome than listed); length of care delay, if applicable (in weeks); how the need for a PA was communicated to the patient (mail or telephone call from insurance company; informed by cancer team or pharmacist; or treatment or test was canceled without notice); patient or caregiver involvement in the PA process (no involvement or handled by care team; call to insurance or management company; call to specialty pharmacy; filed an appeal; involved employer human resources; sought legal counsel; or coordinated between multiple stakeholders); self-rated anxiety (measured on a scale of 0-100, with 0 indicating no anxiety and 100 indicating “overwhelming” anxiety) in respondent’s usual state compared with respondent’s anxiety associated with the PA; changes in trust of the respondent’s care team and insurance provider and of the health care system in general (more trust, no change, or less trust); and overall experience (5-point Likert-style scale, where 1 indicates great, 3 indicates fair, and 5 indicates horrible).

Respondents reporting more than 1 PA were asked to base responses on their most memorable experience. The full survey is available in the eAppendix in Supplement 1. The institutional review board of Memorial Sloan Kettering Cancer Center approved the survey as exempt research; all procedures, including survey design, revision, and recruitment, were conducted in consultation with patient advisors. Written consent was waived by the institutional review board of Memorial Sloan Kettering Cancer Center under 45 CFR 46 Exemption 2, given the anonymous nature and minimal risk of the survey. The findings presented in this cross-sectional study followed the Strengthening the Reporting of Observational Studies in Epidemiology (STROBE) reporting guideline for observational studies.

### Statistical Analysis

The descriptive statistics and univariate tests characterized the survey responses; multivariable analysis, controlling for age, diagnosis, race and ethnicity, and educational level, assessed associations with trust and delays in care. For analysis, outcomes were combined to reflect receipt or nonreceipt of care, owing to the PA. Statistical analysis was performed from June to August 2023. Analyses were conducted using IBM SPSS Statistics, version 27 (IBM Corp), and statistical tests were performed using 2-tailed hypotheses, with an a priori significance level of α = .05.

## Results

Of 178 respondents, most were women (158 [88%]), non-Hispanic White (151 [84%]), college graduates (151 [84%]), and young (18-39 years, 73 [41%]; 40-54 years, 59 [33%]; <65 years, 164 [92%]), with private insurance (151 [84%]) (Table). Breast cancer (78 [44%]), hematologic malignant neoplasms (21 [12%]), and lung cancer (20 [11%]) were the most common diagnoses, with most respondents receiving a diagnosis at stage II or III (79 [44%]) or stage IV (55 [31%]); 41 respondents (23%) reported experiencing a disease recurrence. Nearly one-half of respondents (78 [44%]) received a diagnosis of cancer within 3 years of the survey, 55 (31%) received a diagnosis 3 to 4 years prior to the survey, and 41 (23%) received a diagnosis 5 or more years prior. Respondents were at a variety of stages in their treatment trajectory; 66 (37%) reported that they were undergoing active treatment, 35 (20%) were undergoing hormonal maintenance therapy, 42 (24%) were undergoing other systemic maintenance therapy, and 43 (24%) had completed all treatment (respondents could select >1 response).

**Table.** Demographic and Clinical Characteristics of Respondents

Characteristic	Respondents, No. (%) (N = 178)
Age, y	
18-39	73 (41)
40-54	59 (33)
55-64	32 (18)
≥65	12 (7)
Missing	2 (1)
Gender	
Woman	158 (88)
Man	13 (7)
Nonbinary	1 (1)
Prefer not to respond	4 (2)
Missing	2 (1)
Race and ethnicity	
Asian	10 (6)
Black, non-Hispanic	4 (2)
Hispanic or Latino/a/x	6 (3)
Native American	3 (2)
Native Hawaiian or Pacific Islander	2 (1)
White, non-Hispanic	151 (84)
Other	2 (1)
Did not provide race or ethnicity	2 (1)
Missing	6 (3)
Educational level	
Graduate degree	77 (43)
Bachelor’s degree	65 (36)
Associate’s degree	9 (5)
Some college	20 (11)
High school	5 (3)
Missing	2 (1)
Current household income, $	
<20 000	8 (5)
20 000-39 999	12 (7)
40 000-59 999	15 (8)
60 000-99 999	26 (15)
≥100 000	86 (48)
I prefer not to respond	29 (16)
Missing	2 (1)
Insurance^a^	
Private	151 (84)
Medicare	16 (9)
Medicare Advantage	5 (3)
Medicare supplement	5 (3)
Medicare prescription drug (Part D)	4 (2)
Medicaid	12 (7)
Tricare or other governmental	4 (2)
Missing	2 (1)
Diagnosis^a^	
Breast	78 (44)
Lung	20 (11)
Gastrointestinal	14 (8)
Gynecologic	16 (9)
Hematologic	21 (12)
Sarcoma	10 (6)
Other	24 (13)
Missing	2 (1)
Stage at diagnosis	
Small or localized (stages 0 and I)	32 (18)
Spread to lymph nodes and/or large size (stages II and III)	79 (44)
Spread to other places (metastatic or stage IV)	55 (31)
Liquid tumor or not staged	8 (5)
Missing	5 (3)
Current disease status^a^	
Undergoing active treatment	66 (37)
Undergoing maintenance treatment with hormone therapy	35 (20)
Undergoing other maintenance treatment	42 (24)
Completed all treatment, with no evidence of disease	43 (24)
Disease recurrence, localized	7 (4)
Disease recurrence, metastatic	34 (19)
Missing	4 (2)

^a^ Respondents could select more than 1 response.

Prior authorization was most frequently required for imaging (126 [71%]), intravenous chemotherapy (87 [49%]), surgery (84 [47%]), and radiotherapy (60 [34%]). Most patients reported experiencing PA for the same type of cancer care several times (ie, 2-4 times) (67 [38%]) or many times (ie, ≥5 times) (53 [30%]). Most of the time, the care team (including the pharmacy) informed the patient of the PA (92 [56%]), with 43 (24%) notified by their insurance and 20 (11%) learning after care was canceled without notice. One-half were “surprised” by the PA (88 of 176 [50%]); the remainder (n = 88) were informed by their care team that PA would likely (37 [42%]) or possibly (51 [58%]) be an issue. For most respondents (133 [75%]), the PA experience occurred within the 3 years of the survey and was positively correlated with time since diagnosis (ρ = 0.47; *P* < .001).

Outcomes of the PA were as follows: 112 respondents (63%) reported that their cancer care was approved and given as recommended; 39 (22%) did not receive the care recommended by their oncology team, due to delays that necessitated a new treatment plan or denials; and 17 (10%) received recommended care but paid out of pocket. The remainder experienced other outcomes, including having care written off by the hospital and/or changing insurances. Of the 39 respondents who did not receive the recommended care, 26 (67%) did not receive it because their insurance completely denied the PA, and 13 (33%) did not receive it because the PA-related delay necessitated a different treatment plan (even though the original treatment was ultimately approved). Of the 17 respondents who paid out of pocket, 9 (53%) reported receiving a “surprise” bill (vs knowing they would have to pay out of pocket).

Most patients (123 [69%]) reported a delay in care due to PA; of those with delayed care, 90 (73%) reported a delay of 2 or more weeks, and one-third (40 [33%]) reported a delay of 1 month or longer. The Figure shows delays by treatment type. Delays did not vary by demographic, clinical, insurance, or treatment factors. but there was a positive association between length of reported delay and the recency of the PA (ρ = 0.20; *P* = .007).

**Figure.**
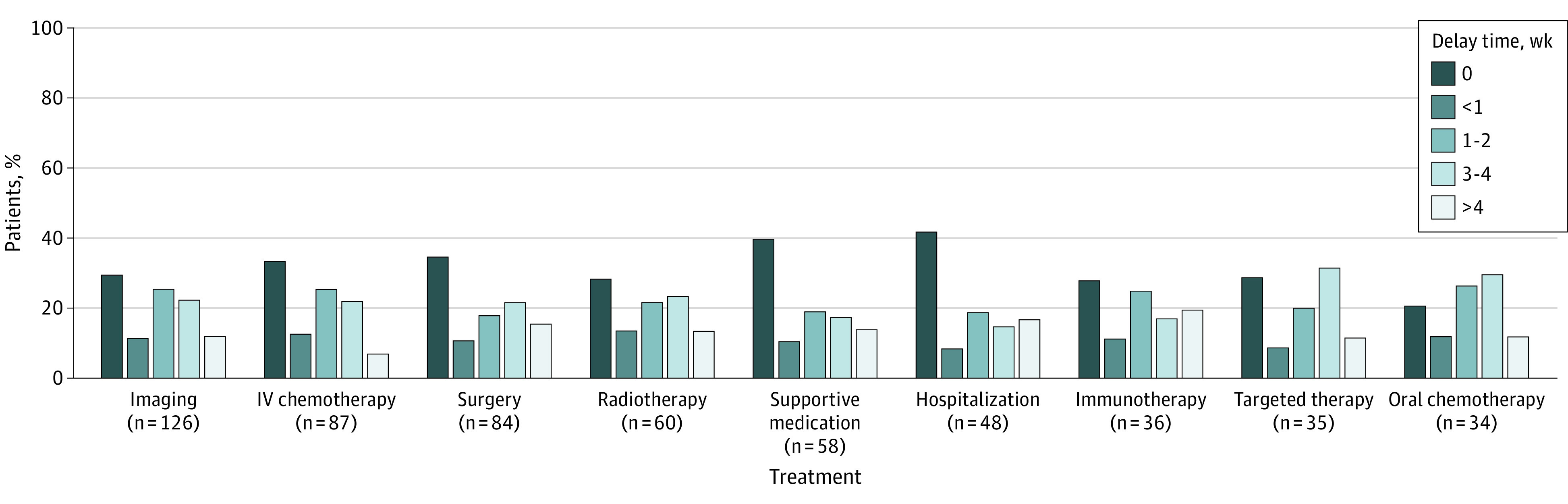
Delays by Treatment Type IV indicates intravenous.

Most respondents (119 [67%]) had to personally become involved in the PA process by calling their insurance (94 [53%]) or filing an appeal (45 [25%]); 58 respondents (33%) reported that their health care team handled it completely. Most patients (116 [65%]) spent less than 5 hours on PA, but 35 patients (20%) and/or their caregivers spent 11 or more hours dealing with PA issues. Time spent dealing with the PA was positively associated with delay length (ρ = 0.28; *P* < .001).

Overall, the PA experience was rated as bad (70 [40%]) or horrible (55 of 174 [32%]) by most respondents; worse ratings were associated with delay length (ρ = 0.36; *P* < .001) and time spent on PA (ρ = 0.42; *P* < .001). Self-reported PA-related anxiety was higher than respondents’ usual level of anxiety (mean [SD] score, 74.7 [20.2] vs 37.5 [22.6]; *P* < .001] and was correlated with delay length (ρ = 0.16; *P* = .04), time spent on PA (ρ = 0.27; *P* < .001), and overall PA experience (ρ = 0.34; *P* < .001).

PA was associated with patient trust; 31 respondents (18%) trusted their cancer team less, 159 (89%) trusted their insurance company less, and 148 (83%) trusted the health care system less. Less trust did not vary by demographic, clinical, insurance, or treatment factors.

In logistic regression models that considered respondent age, race and ethnicity, educational level, and diagnosis, patient involvement in the PA process (vs health care team handling the PA process completely) was associated with increased odds of distrusting their insurance company (β = 6.0; 95% CI, 1.9-19.2), distrusting the health care system (β = 3.3; 95% CI, 1.4-8.1), and rating the PA experience as negative (vs neutral or positive; β = 6.6; 95% CI, 3.1-14.3). Having to file an appeal was associated with 3.0 increased odds (95% CI, 1.2-7.3 increased odds) of experiencing a delay in care.

## Discussion

This survey-based cross-sectional study highlights the adverse association of PA with outcomes among a convenience sample of patients with cancer; 22% of the sample reported that they were prevented from receiving the care recommended by their treatment team because of PA. Most respondents experienced a delay in recommended oncology care because of PA, and delays were associated with increased anxiety, a negative perception of the PA process, and patient administrative burden.

Prior authorization–related delays in cancer care are common; in 1 survey, nearly all responding oncologists (93%) said that PA delayed life-saving treatments for their patients, with 31% estimating delays exceeding 5 days.^7^ Similar findings have been reported in pediatric oncology, with nearly two-thirds of clinicians reporting delays or changes to planned chemotherapy or supportive care regiments because of PA.^3^ The PA process at academic radiotherapy centers is estimated to cost an additional $40 million or more in clinician time; 85% of services were ultimately approved, suggesting that PA only served to delay treatment and waste time for both patients and clinicians.^8^ These results are similar to findings in the pediatric setting, in which 99% of 137 PAs for medication were approved after clinician involvement.^9^ Even if PA rejections appropriately restrict a limited amount of non–evidence-based care, the process frequently requires additional clinician time and overcoming logistic hurdles that may impair the quality of care for many more patients by delaying timely treatment.^9,10,11^ Lifting restrictive PA practices has been associated with guideline-concordant care in other specialties.^12^

Efforts to create national health policy solutions that streamline PA and make the process more transparent have been a major lobbying effort of large oncology societies.^13,14^ Pending bicameral, bipartisan legislation via the Improving Seniors’ Timely Access to Care Act would establish regulations on the quality and timeliness of PA in the Medicare Advantage population and would require real-time decisions for items and services that are routinely approved.^15^ Despite passing unanimously in the US House of Representatives in 2022^16^ and acquiring 52 cosponsors in the Senate,^17^ the bill has been stalled. In the meantime, the Centers for Medicare & Medicaid Services acted directly by issuing a final rule in April 2023 aimed at improving PA processes within the Medicare Advantage population^18^ by 2024.

To our knowledge, this study is the first to ask oncology patients about their experience with PA. Understanding the patient perspective is a vital component of efforts to refine the PA process, given that patients (and their families) are ultimately financially responsible for resultant medical bills and often must take on the administrative burdens of PA while simultaneously managing the physical and psychosocial effects of their disease and treatment.^19^ Prior studies have suggested that additional burdens are associated with delayed or forgone care and are more likely to affect those who are already at risk for compromised or fractured care, including Black or African American, disabled, female, and low-income patients.^20,21^ Research also shows that PA may place disproportionate limitations to access on vulnerable populations, patients of Asian descent, and those with Medicare Advantage plans.^22,23,24^ Dealing with PA issues adds an extra layer of stress, which is known to increase anxiety and can worsen treatment-related and disease-related symptoms and adverse effects.^25^

### Limitations

Our findings should be interpreted in the context of study limitations, as the use of convenience sampling likely produced a sample enriched to discuss negative experiences. Furthermore, young non-Hispanic White women were overrepresented in the sample, which both biases the findings and limits their generalizability. Because this was an anonymous survey using online recruitment, we were unable to determine an accurate response rate or distinguish characteristics between responders and nonresponders. Although we included a screening question to determine eligibility, we cannot confirm with certainty that the patient (vs a caregiver) completed the survey. Nearly one-fourth of the sample had completed treatment, which may subject our findings to recall bias, although this was likely mitigated to an extent by our findings that trust (in the care team, health insurance company, and health care system), overall experience, and delays were not associated with treatment completion. The insurance-specific reasons for PA delay or denial were outside the scope of this patient-facing survey, and the downstream oncologic outcomes were similarly not assessed in the survey. In addition, patient self-reported anxiety and experiences are, by definition, subjective and complex and thus hard to rigorously categorize. Notwithstanding these limitations, research into the patient experience in oncology is vital to improving patient-centered care and is, to date, lacking in the emergent body of literature addressing PA. Future research can build on our findings to focus on downstream oncologic outcomes and an in-depth exploration of patient experiences in larger, more diverse samples.

## Conclusions

Focusing on patient experiences with PA highlights a missing perspective in policy discussions and suggests another potential factor associated with eroding trust in the health care system.^26^ Streamlining the PA process is key to optimizing the quality of care delivered and improving patients’ experience with cancer care. Policy interventions will be necessary to reform the PA process, as will advocacy efforts at the patient, clinician, and hospital level.^13,15^

## Notes

Supplement 1. **eAppendix.** The Patient Experience With Prior Authorization for Cancer Treatment

Supplement 2.
Data Sharing Statement
