# Supplementary material for: The Patient Experience of Prior Authorization for Cancer Care
Source: JAMA Netw Open. 2023 Oct 18;6(10):e2338182. doi: 10.1001/jamanetworkopen.2023.38182 (PMC10585404; doi:10.1001/jamanetworkopen.2023.38182)
Supplement: Supplement 1. — eAppendix. The Patient Experience With Prior Authorization for Cancer Treatment [file jamanetwopen-e2338182-s001.pdf]

Supplementary Online Content

Chino F, Baez A, Elkins I, Aviki EM, Ghazal LV, Thom B. The patient experience of prior authorization for cancer care. *JAMA Netw Open.* 2023;6(10):e2338182. doi:10.1001/jamanetworkopen.2023.38182

eAppendix. The Patient Experience With Prior Authorization for Cancer Treatment

This supplementary material has been provided by the authors to give readers additional information about their work.

© 2023 Chino F et al. *JAMA Network Open.*

eAppendix. The Patient Experience With Prior Authorization for Cancer Treatment

Prior authorization is the process through which a medical provider (or you, in some cases) must obtain approval from your health insurance before moving ahead with a particular treatment, procedure, test, or medication as prescribed by your physician.

This survey was designed to understand your experience with prior authorization for cancer care. It has 33 questions and will take less than 10 minutes to complete. It is completely anonymous and voluntary; nobody will seek to identify you from your responses. You can skip any question that makes you uncomfortable and you can exit the survey at any time.

If you have questions regarding this survey, please contact the Primary Investigator at Memorial Sloan Kettering, Dr Fumiko Chino (chinof@mskcc.org).

Are you an adult (18yrs or older) who has had personal experience with prior authorization during cancer treatment?

◯ Yes ◯ No

Sorry, you can not take this survey unless you are an adult who has had personal experience with prior authorization for cancer treatment. Thank you for your interest! Have a great day!

---

1. For which cancer treatment/service(s) have you experienced prior authorization? [mark all that apply]

- ☐ Surgery
- ☐ IV Chemotherapy (chemotherapy you receive through an IV at the doctor's office or via a pump at home)
- ☐ Oral Chemotherapy
- ☐ Radiation therapy
- ☐ Immunotherapy
- ☐ Targeted therapy (for specific mutations in the tumor like EGFR or ALK)
- ☐ Hormone therapy
- ☐ Hospital stays
- ☐ Imaging studies (for example: CT, PET/CT, MRI)
- ☐ Supportive Medications (for example: medication for pain, nausea, nutrition, blood thinners)
- ☐ Physical or Occupational Therapy
- ☐ Acupuncture or other complementary therapies
- ☐ Medical equipment or supplies
- ☐ Other (fill in blank)

If other, please indicate here

____

---

2. How often have you experienced issues requiring prior authorization for the same type of cancer treatment/service? [For example: MRI imaging that needs prior authorization each time ordered or pain medication that needs a new prior authorization every year.]

- ◯ Just Once
- ◯ Several times (2-4)
- ◯ Many times (5 or more)
- ◯ Every single time

© 2023 Chino F et al. *JAMA Network Open.*

3. For which cancer treatment/service was the prior authorization process the MOST memorable?	◯ I only experienced the prior authorization process once<br>◯ Surgery<br>◯ IV Chemotherapy (chemotherapy you receive through an IV at the doctor's office or via a pump at home)<br>◯ Oral Chemotherapy<br>◯ Radiation<br>◯ Immunotherapy<br>◯ Targeted therapy (for specific mutations in the tumor like EGFR or ALK)<br>◯ Hormone therapy<br>◯ Hospital stays<br>◯ Imaging studies (CT, PET/CT, MRI etc)<br>◯ Supportive Medications (pain, nausea, nutrition, blood thinners etc)<br>◯ Physical or Occupational Therapy<br>◯ Acupuncture or other complementary therapies<br>◯ Medical equipment or supplies<br>◯ Other ___________(fill in blank)
If other, please indicate here	__________________________

For questions 4-10, please answer regarding the most memorable experience you had with prior authorization as noted above.

4. When did this process of prior authorization occur?	◯ Over 20 years ago (before 2002)<br>◯ 11-20 years ago (between 2002-2011)<br>◯ 5-10 years ago (between 2012-2016)<br>◯ 3-4 years ago (between 2017-2019)<br>◯ 2020<br>◯ 2021<br>◯ 2022
5. How did you first find out that prior authorization was necessary for your cancer care?	◯ I got a letter in the mail<br>◯ I got a phone call from my insurance (or their management company)<br>◯ My cancer team notified me<br>◯ The pharmacist told me when I went to pick up the medication or when I tried to reorder it<br>◯ My treatment or test was cancelled without notice<br>◯ Other (fill in blank)
If other, please indicate here	__________________________

© 2023 Chino F et al. *JAMA Network Open.*

6. What was the resolution of the prior authorization?	◯ The treatment was APPROVED by my insurance, and I got the cancer care originally recommended<br>◯ The treatment was APPROVED, but I got a different cancer care plan than the original because we couldn't wait<br>◯ The treatment was NOT approved, but I still got the cancer care originally recommended because I decided it was worth it to pay out-of-pocket for it<br>◯ The treatment was NOT approved, I got the cancer care originally recommended, and I ended up with a surprise bill after the fact<br>◯ The treatment was NOT approved, and I got a different cancer care plan<br>◯ Other (fill in blank)
If other, please indicate here	______________________________
7. Did prior authorization delay your cancer care?	◯ No<br>◯ Yes, by less than 1 week<br>◯ Yes, by 1 week<br>◯ Yes, by 2 weeks<br>◯ Yes, by 3 weeks<br>◯ Yes, by 4 weeks<br>◯ Yes, by more than a month<br>◯ Yes, by several months
8. Did your doctor tell you about the prior authorization process IN ADVANCE?	◯ They told me that it would likely be an issue<br>◯ They warned me that it could possibly be an issue<br>◯ No, it was a surprise
9. Did you (or your caregiver) personally get involved in the prior authorization process? [mark all that apply]	☐ No, it was completely handled by my cancer team<br>☐ I/We had to call my insurance<br>☐ I/We had to call my healthcare utilization management company<br>☐ I/We had to call the specialty pharmacy<br>☐ I/We had to file an appeal<br>☐ I/We had to get someone from my work (for example: human resources or an ombudsman) involved<br>☐ I/We had to get a lawyer involved<br>☐ I/We had to coordinate between several different stakeholders
10. How many hours did you (or your caregiver) spend in the last year dealing with issues related to prior authorization:	◯ Less than 1 hour<br>◯ 1-5 hours<br>◯ 6-10 hours<br>◯ 11-20 hours<br>◯ Over 20 hours

For questions 11-19, please answer regarding the overall experience you have had with prior authorization.

© 2023 Chino F et al. *JAMA Network Open.*

11. How did the prior authorization process make you feel? [mark all that apply]	☐ Neutral<br>☐ Frustrated<br>☐ Annoyed<br>☐ Happy<br>☐ Sad<br>☐ Angry<br>☐ Anxious<br>☐ Fearful<br>☐ Invigorated<br>☐ Defeated<br>☐ Exhausted<br>☐ Other (fill in blank)
If other, please indicate here	____________________________
12. How would you rate your anxiety when dealing with issues of prior authorization from 0-100? [0=no anxiety, 100=overwhelming anxiety]	no anxiety — moderate anxiety — overwhelming anxiety<br>*(Place a mark on the scale above)*
13. How would you rate your anxiety on a regular basis (unrelated to prior authorization) from 0-100? [0=no anxiety, 100=overwhelming anxiety]	no anxiety — moderate anxiety — overwhelming anxiety<br>*(Place a mark on the scale above)*
14. How could your cancer team have better prepared you for the prior authorization process? [mark all that apply]	☐ They should warn people when they order the test/treatment<br>☐ They should explain the process better<br>☐ They should give me more frequent updates on the appeals process<br>☐ They should guide me on how to talk to my health insurance<br>☐ They should reassure me that if this treatment/test isn't approved they can give me another<br>☐ They couldn't have prepared me better<br>☐ I don't know<br>☐ Other (fill in blank)
If other, please indicate here	____________________________
15. Do you think the prior authorization process made a difference to your cancer outcomes (i.e., how successful the treatment was or your long term cancer survival and/or side effects)?	○ Yes, it made my cancer outcomes worse<br>○ Yes, it made my cancer outcomes better<br>○ No
16. What was your overall experience with prior authorization?	○ It was great<br>○ It was good<br>○ It was fair<br>○ It was bad<br>○ It was horrible
17. How did the prior authorization process affect how you feel about your cancer team?	○ It made me trust them more<br>○ It did not change my opinion<br>○ It made me trust them less

© 2023 Chino F et al. *JAMA Network Open.*

18. How did the prior authorization process affect how you feel about your insurance company?	◯ It made me trust it more<br>◯ It did not change my opinion<br>◯ It made me trust it less
19. How did the prior authorization process affect how you feel about the healthcare system in general?	◯ It made me trust it more<br>◯ It did not change my opinion<br>◯ It made me trust it less
20. In days, what is the longest time period that prior authorization has ever delayed your cancer care? [enter 0 for no delay]	________________________________
21. Please tell us anything else you would like to share about the process of prior authorization:	________________________________
22. When were you diagnosed with cancer?	◯ Over 20 years ago (before 2002)<br>◯ 11-20 years ago (between 2002-2011)<br>◯ 5-10 years ago (between 2012-2016)<br>◯ 3-4 years ago (between 2017-2019)<br>◯ 2020<br>◯ 2021<br>◯ 2022
23. What type of cancer were you diagnosed with? [mark all that apply]	☐ Breast<br>☐ Lung<br>☐ Gastrointestinal (for example: Colorectal, Anal, Pancreas, Esophagus, Stomach, Liver)<br>☐ Prostate<br>☐ Gynecological (for example: Ovary, Uterus, Cervix, Vulva)<br>☐ Hematological (for example: Leukemia, Lymphoma, Multiple Myeloma)<br>☐ Sarcoma<br>☐ Head and Neck<br>☐ Other (fill in blank)
If other, please indicate here	__________________________
24. What stage was the cancer at initial diagnosis?	◯ Small/localized (Stage 0 and I)<br>◯ Spread to lymph nodes and/or large size (Stage II and III)<br>◯ Spread to other places (metastatic or Stage IV)<br>◯ Liquid tumor or not staged

© 2023 Chino F et al. *JAMA Network Open.*

25. What is your current disease status? [mark all that apply]	☐ Undergoing active treatment<br>☐ Undergoing maintenance treatment with hormone therapy (for example: Lupron for breast or prostate cancer, tamoxifen or aromatase inhibitors for breast cancer)<br>☐ Undergoing maintenance treatment with some other systemic therapy (for example: ongoing Herceptin/Trastuzumab for breast cancer, Tagrisso/Osimertinib for lung cancer, or Lynparza/Olaparib for ovarian cancer)<br>☐ Completed all treatment, with no evidence of disease<br>☐ Disease recurrence, localized<br>☐ Disease recurrence, metastatic
26. What treatments have you received (or are currently receiving) for cancer? [mark all that apply]	☐ Surgery<br>☐ IV Chemotherapy (chemotherapy you receive through an IV at the doctor's office or via a pump at home)<br>☐ Oral Chemotherapy<br>☐ Radiation therapy<br>☐ Immunotherapy<br>☐ Targeted therapy (for specific mutations in the tumor like EGFR or ALK)<br>☐ Hormone therapy<br>☐ Other (fill in blank)
If other, please indicate here	________________________________
27. How old are you?	◯ 18-39<br>◯ 40-54<br>◯ 55-64<br>◯ 65-74<br>◯ 75 and older
28. What is your Gender?	◯ Woman<br>◯ Man<br>◯ Transgender woman<br>◯ Transgender man<br>◯ Non-binary<br>◯ Another gender (fill in blank)<br>◯ I prefer not to respond
If another gender, please indicate here	________________________________
29. What is your race? [mark all that apply]	☐ White<br>☐ Black/African American<br>☐ Asian<br>☐ Native Hawaiian and/or Pacific Islander<br>☐ Native American<br>☐ Another race (fill in blank)
If other, please indicate here	________________________________
30. What is your ethnicity?	◯ Hispanic/Latino/Latinx<br>◯ Not Hispanic/Latino/Latinx

© 2023 Chino F et al. *JAMA Network Open.*

31. What is your health insurance type? [mark all that apply]	☐ Private Insurance (through you or your spouse's employer)<br>☐ Private Insurance (through the marketplace)<br>☐ Medicare<br>☐ Medicare Advantage<br>☐ Medicare Supplement Insurance (Medigap)<br>☐ Medicare Prescription Drug Plan (Part D)<br>☐ Medicaid<br>☐ Emergency Medicaid<br>☐ Tricare or other government insurance<br>☐ No health insurance/charity care
32. What is your highest level of education?	◯ Less than high school graduate<br>◯ High school graduate<br>◯ Some college<br>◯ Associate's degree<br>◯ Bachelor's degree<br>◯ Graduate/professional degree
33. What is your current household income?	◯ < $20,000<br>◯ $20,000-39,999<br>◯ $40,000-59,000<br>◯ $60,000-99,000<br>◯ $100,000 or above<br>◯ I prefer not to respond

© 2023 Chino F et al. *JAMA Network Open.*
