# Supplementary material for: The Patient Experience of Prior Authorization for Cancer Care
Source: JAMA Netw Open. 2023 Oct 18;6(10):e2338182. doi: 10.1001/jamanetworkopen.2023.38182 (PMC10585404; doi:10.1001/jamanetworkopen.2023.38182)
Supplement: Supplement 2. — Data Sharing Statement [file jamanetwopen-e2338182-s002.pdf]

Data Sharing Statement

Chino. The Patient Experience of Prior Authorization for Cancer Care. *JAMA Netw Open*. Published October 18, 2023. doi:10.1001/jamanetworkopen.2023.38182

Data
Data available: No

Additional Information
Explanation for why data not available: Our institutional IRB standards do not include data sharing and (although anonymous) the nature of survey comments relayed are highly personal
